# Sex Differences in Cognitive-Motor Dual-Task Training Effects and in Brain Processing of Semi-Elite Basketball Players

**DOI:** 10.3390/brainsci13030443

**Published:** 2023-03-04

**Authors:** Stefania Lucia, Merve Aydin, Francesco Di Russo

**Affiliations:** 1 Department of Movement, Human and Health Sciences, University of Rome “Foro Italico”, 00135 Rome, Italy; 2 Santa Lucia Foundation IRCCS, 00179 Rome, Italy

**Keywords:** cognitive-motor dual-task training, sex differences, ERP, cognitive neuroscience, anticipation, sport

## Abstract

In the current study, we aimed at evaluating the possible sex differences in cognitive-motor dual-task training (CMDT) effects on the sport and cognitive performance of semi-elite basketball athletes. Moreover, we investigated the CMDT effects on proactive brain processing using event-related potential (ERP) analysis. Fifty-two young basketball athletes (age 16.3 years) were randomly assigned into an experimental (Exp) group performing the CMDT, and a control (Con) group executing standard motor training. Before and after a 5-week training intervention, participants’ motor performance was evaluated using dribbling tests. Cognitive performance was assessed by measuring response time and accuracy in a discrimination response task (DRT). Brain activity related to motor and cognitive preparation was measured through the Bereitschaftspotential (BP) and the prefrontal negativity (pN) ERP components. The CMDT involved the simultaneous execution of dribbling exercises and cognitive tasks which were realized using interactive technologies on the court. Results showed that both groups had some enhancements from pre- to post-tests, but only the Exp group enhanced in the dribbling exercise. In the DRT after the CMDT, females performed faster than males in the Exp group. All groups, except for the Con group of males, performed the DRT more accurately after the training. According to the ERP results, in the Exp group of males and in Exp and Con group of females, we found an increase in pN amplitude (associated with better accuracy); in the Exp group of females and in Exp and Con group of males, we found an increase in BP (associated with better response time). In conclusion, the present study endorsed the efficacy of the proposed CMDT protocol on both the sport and cognitive performance of semi-elite basketball players and showed that the neural basis of these benefits may be interpreted as sex-related compensatory effects.

## 1. Introduction

An efficient training methodology is fundamental for professional athletes and thanks to the union of sport and cognitive sciences, new training protocols have been developed to improve both physical and cognitive performance. A type of training that is receiving increasing interest is the so-called cognitive-motor dual-task training (CMDT) in which physical and cognitive trainings are required within the same task. CMDT has been proven more effective than motor or cognitive training alone to improve cognitive-motor performance in different age groups e.g., [1,2]. In Lucia et al. [3,4], we showed that a five-week basketball-specific CMDT protocol may improve both sport and the cognitive performance of semi-professional basketball players more than motor training alone. In addition, these studies showed that the benefit produced by the CMDT may be due to improved brain processing, especially in task preparation [3] and decision-making [4] functions. These studies were done on male athletes only [3] or did not distinguish between sexes [4] which is an important moderator of cognitive and sport performance.

Sex differences are found in many cognitive tasks, sometimes favouring females in tests of writing, language fluency, learning, and memory, and sometimes favouring males in visuospatial tasks or problem-solving tests [5]. Indeed, some studies have focused on sex differences in basketball players with attention on cognition. For example, Millslagle [6] examined the recognition accuracy using a perceptual-cognitive paradigm, in which athletes observed images of structured and unstructured basketball game situations. The study found that for the male players, situation recognition was better than for the female players. Weigelt and Memmert [7] investigated mental rotation (tasks adapted for basketball) and observed that males solved more items than females independently from expertise level. Milley and Ouellette [8] studied the shifting to an external focus of attention with imagery techniques to improve free-throw scores but without testing for possible sex differences. El Moutaraji et al. [9] in athletes found that even though sexes did not differ in terms of visual perception and information speed processing, males were faster than females in motor and choice response time. Considering physical basketball performance, there are some studies on sex differences. For example, according to their chronological age and professional league, men are greater in jumping ability [10] which might be an advantage for them to shoot the ball into the basket or to take a rebound with respect to females. Men prefer 3-point shots whereas women prefer 2-point shots [11]. In semi-professional basket players, Scanlan et al. [12] found that during matches, males perform more dribbling actions than females, but on the other hand, females run more than males. Thus, there are studies on sex differences in cognition and on the technical aspects of the game but, to our knowledge, no study has investigated the neural basis of sex differences in basketball players, as was carried out by Bianco et al. [13] using the event-related potential (ERP) method to study cognitive and motor preparation in the pre-stimulus phase of visuomotor tasks. The results confirmed the sex-related speed/accuracy trade-off in which males were faster and females were more accurate in visuomotor cognitive tasks [14,15]. Furthermore, they found a similar relationship between the underlying brain functions with larger motor preparation in males and larger cognitive preparation in females. Motor preparation has been indexed by the Bereinshaftpotential (BP) originating in premotor brain areas and associated with motor readiness preceding any voluntary movement [16]. Cognitive preparation has been indexed by the prefrontal negativity (pN), originating in the prefrontal cortex and associated with top-down attentional and inhibitory control [17].

Considering the found sex differences in cognitive and motor performance, it is important that sports coaches optimize the type of individual training (such as the CMDT) as a function of the athlete’s sex, taking into account the athlete’s physical and/or cognitive needs (i.e., stimulating more response speed in females and more response accuracy in males). If there are any differences, the coach could use them to differentiate training protocols on dribbling, for example by changing the exercise space or proposing cognitive exercises with a different stimulation time. For this reason, the study aims to investigate possible sex differences in the CMDT effects on semi-elite basketball players using the same methodology as Lucia et al. [3]. Specifically, the difference between males and females in preparatory brain functions indicated by Bianco et al. [13] persuaded us to investigate these differences in athletes. The dribbling execution times for simple and complex actions were chosen as the sports tests, the response speed and accuracy in a DRT as the cognitive test, and the BP and the pN ERP components as indexes of motor and cognitive brain preparation. 

Considering previous findings, we expected to observe a faster performance in males than females in the dribbling tests because males have more practice with these actions [12]. Regarding the CMDT-related effect, we expected an improvement in both sexes, confirming the results of Lucia et al. [3,4] and extending them to female athletes. In terms of behavioural results in the cognitive task, we expected to confirm Bianco et al. [13] outcomes showing a faster response time in males than females and higher response accuracy in females than males. In addition, we hypothesized differential sex-related effects of the CMDT that could compensate for the weakness of each sex (response accuracy in males and response time in females). Finally, we expect to confirm the found sex differences in the brain functions associated with task preparation [13] and to also find differential CMDT effects in preparatory brain activity. Specifically, the CMDT may stimulate motor processing more in females and cognitive processing more in males. If confirmed, these results could contribute to our knowledge of sex differences in cognitive function and sport performance. In addition, the results might be applied to the optimization of the CMDT protocol according to the athlete’s sex.

## 2. Materials and Methods

### 2.1. Participants

The sample size was determined with the G*power 3.1.9.7 software [18], estimating effect size from Cohen’s f statistics. We set an effect size f at 0.25 based on the minimum significant partial eta squared effect size obtained in [3]. As traditionally done, the α level was set at 0.05, and the desired power (1 − β) at 0.95 in order to keep α and β levels equal [18]. This calculation estimated a minimum sample size of 52; therefore, 52 young semi-elite basketball players (28 females mean age 16.32 ± 1.0, 24 males, mean age 16.33 ± 1.1 years) were enrolled in the study. All athletes were members of the sport society “Stella Azzurra Basketball” of Rome and were part of Under-18 teams. The following inclusion criteria were considered: naïve about the aim of the study, absence of any neurological and psychiatric disorders, not on medication during the experimental sessions, normal or corrected-to-normal vision, and being fully right-handed (Edinburgh handedness inventory [19]). Participants were further required to be actively involved in basketball practice and to have at least 6 years of formal training in basketball. Both parents of all participants gave their informed consent before participating in this study in accordance with the Declaration of Helsinki after approval by the local ethical committee in the institutional review board of the University of Rome “Foro Italico” (protocol code CARD-74/2020).

### 2.2. Procedure

Athletes were pseudo-randomly assigned to two groups of 26: the experimental (Exp) and the control (Con) group using a binary random-number table. In each group, males and females were analysed separately (two subgroups of 12 males and two subgroups of 14 females). Groups did not differ in age, education, socioeconomic status, or expertise. According to a 2 × 2 analysis of variance (ANOVA), Swann classification [20] revealed no statistically significant differences (F_(1,48)_ < 1) in terms of expertise between groups. The average score was 3.5 ± 0.3 corresponding to the semi-elite level.

Training and testing were the same adopted by Lucia et al. [3,4]. The Con group was trained for five weeks, seven times a week: one day for a basketball match, and six times a week for standard basketball training with group basketball training (3 h) and two standard individual training sessions (30 min); this included physical and technical exercises only. The Exp group performed the same training schedule, but the two individual sessions of 30 min were done using the CMDT described below. Before and after the training, all participants completed specific tests for assessing basketball performance and a cognitive task performed during electroencephalographic (EEG) recording. Pre- and post-tests were executed 1–2 days before and after the treatment (basketball performance tests two days before and after, cognitive and EEG tests one day before and after). 

#### 2.2.1. Motor Training

**Figure 1:**
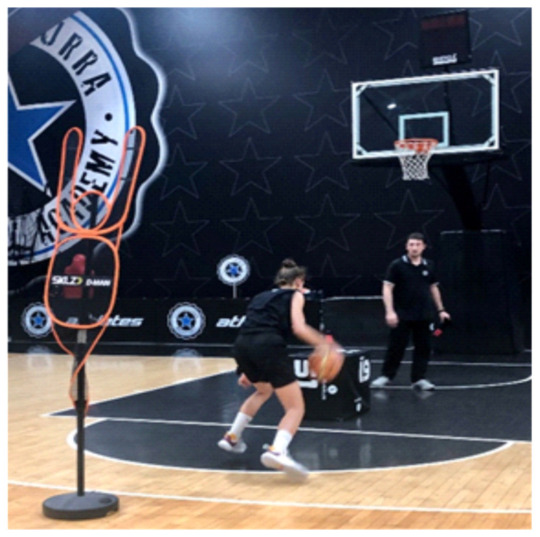
Standard individual training session with the coach.

A typical individual training session is divided into three phases: activation, central, and final phases, as described in the basic training fundamental. During each training, the coach concentrates on dribbling with a different hand, speed, and direction changes resulting in shots (Figure 1). To practice the essential that enables the athlete to keep and sustain the advantage over the opponent, exercises were established in routines on the dribble to stimulate body movements with the ball using harmony and power [21]. The training was conducted with one ball or two balls at the same time. Below, are described three sample exercises of activation, central and final phases are described as follows (see also Supplementary Video Material):

1. The athlete was activated from a stationary position, in front of a cone, and alternated dribbles with the right hand and left hand, under the legs, behind the back, in/out front, and side wave with the right and left hand.
2. The athlete uses the cone positioned at 5.5 m as a reference. He sprints by dribbling with his right hand up to the cone and then performs backward dribbling with his right hand until he reaches the starting position where he performs a front-hand change and restarts with his left hand. Each time he/she returns to the starting position, the athlete uses different hand changes to restart (e.g., frontal, under the legs, behind the back).
3. The athlete uses two positioned hurdles (refer to Figure 1) and performed the exercise similar to the previous one but using different distances in retreat depending on the cone and the different hand changes.

For males, #7 standard National Basketball Association (NBA) and International Basketball Federation (FIBA) balls were used (75–78 cm circumference, 567–650 g weight). For females, the FIBA and WBNA regulation ball size #6 was used (72–74 cm circumference and 500–540 g weight).

#### 2.2.2. Cognitive-Motor Dual-Task Training (CMDT)

The experimental protocol included a CMDT that demanded the concurrent finalization of motor and cognitive exercises. The training aimed to enhance functional abilities and cognitive functions. Short routines of exercises were planned to simultaneously stimulate several cognitive functions as well as technical fundamentals. For instance, participants completed task sequences that “scattered” or reversed the acquired order in order to train the inhibition of automatic responses and challenge decision making [3,4]. Moreover, they were instructed to learn various stimulus-response connections and then to switch between them in response to the shifting of external cues while also producing a motor response. 

**Figure 2:**
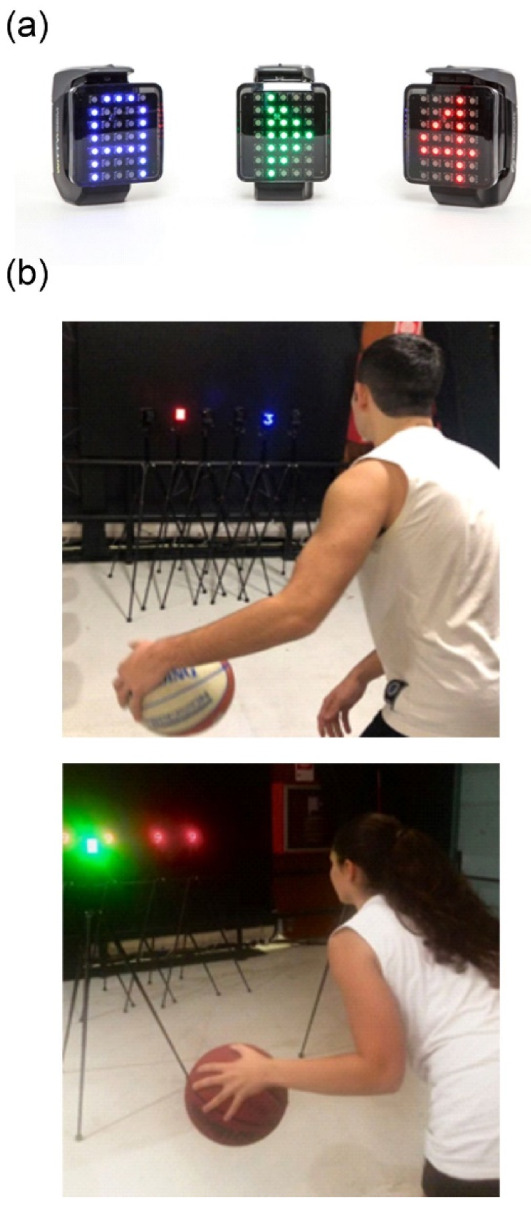
(**a**) Interactive display devices showing some of the possible colour, letter, or number outputs; (**b**) frames showing snapshots of the CMDT during 2 of 6 exercises.

The Witty-SEM system was utilized to conduct the training (Microgate, Bolzano, Italy). This system has a LED screen displaying symbols of different colours that can interact with athletes thanks to proximity sensors. A picture of these devices is shown in Figure 2a. During this training, the Exp group was asked to perform six CMDT exercises involving agility, precision, and control in dribbling and simultaneously train cognitive functions such as anticipation, discrimination, working memory, and decision-making (Figure 2b). An accurate description of the single exercises is reported in [3] and in the Supplementary Video Material. Below are three exercises as examples:

1. To activate, the athlete must close only the “Witty-SEM” with the “Blue 7” as quickly as possible by performing a different hand change each time (e.g., frontal, under the legs, behind the back).
2. The athlete is positioned in front of two devices. “A” is green all the time, while “B” changes colour and configuration. The athlete must perform different hand changes depending on the colour of the “B” photocell (e.g., blue-under the legs; red-behind the back) and by dribbling close to the “A”.
3. The athlete must be ready to discriminate the photocell with the different configurations within a few milliseconds while performing hand changes (e.g., frontal). Once he/she detects the different devices, he/she must sprint-dribble and close it and then perform backward dribbles to return to the starting position and continue with the exercise.

#### 2.2.3. Basketball Performance Tests

Five tests on dribbling, one of the fundamentals of basketball, were used to assess the effects of the treatment on basketball performance. These tests were created based on previous studies that test players while sprinting and dribbling at the same time [22,23,24]. Athletes were asked to dribble along the side strip of the basketball court and change hands as fast as possible whenever they saw a cone placed on the floor. The cones were 5.5 m apart. 

The hand changes were crossover, double crossover, between the legs, crossover and between the legs, between the legs and behind the back. Since the hand changes involve different technical difficulties, they can be divided into single changes dribbling (crossover and between the legs) and multiple changes dribbling (double crossover, crossover and between the legs, behind the back). Exercise completion times were measured for each test, and subsequently, the average score for each circuit was calculated for the two difficulty levels. These tests were selected because athletes have to run and dribble simultaneously, balancing sex strengths/weaknesses [12].

#### 2.2.4. Cognitive Test

The cognitive test consisted of a discriminative response task (DRT), based on the Go/No-go paradigm, and was performed during electroencephalogram (EEG) recording in the Cognition and Action Neuroscience Laboratory at the University of Rome “Foro Italico”. This task was used to test the CMDT effect on general cognitive performance using a paradigm largely used in the literature, e.g., [17] that is well-suited to obtain reliable pre-stimulus ERP. Participants were assessed in a low-lit, sound-attenuated room after the EEG cap was set to the scalp. They were positioned in front of a computer screen placed 114 cm from their eyes with their right index finger on a push button board. The fixation point was a yellow circle with a diameter of 0.15° on a black background in the centre of the screen throughout the whole experimental session. Four visual stimuli (i.e., square configurations subtending 4 × 4° and made by vertical and/or horizontal bars) were randomly visualized on the screen for 250 ms with equal probability (*p* = 0.25); the stimulus–onset asynchrony varied from 1 to 2 s to prevent stimulus prediction and ERP overlaps with previous and following stimuli. Participants had to push the button with their right index finger as soon as possible only when the designated target stimuli “go” appeared (two out of four times) on the screen (*p* = 0.5), and not respond when non-target stimuli “no-go” appeared (*p* = 0.5); response time and accuracy were analysed. The order of presentation of the four stimuli was randomized between runs. The duration of each run was 2 min interleaved with 30 s pauses. Ten runs were performed allowing us to obtain 400 trials for each stimulus category in approximately 40 min.

##### Behavioral Data

Mean response times (RTs) for correct trials were calculated for each participant. Accuracy was calculated as the percentage of false alarms (FA) i.e., erroneous responses to non-target stimuli (commission errors).

##### EEG Recording

The EEG was recorded using the Recorder 1.2 software and three BrainAmp amplifiers, two of them connected to 64 active sensors actiCAP; data were processed using the Analyzer 2.2.2 package (all by Brain Products GmbH, Gilching, Germany). Electrodes were mounted according to the 10–10 international system and referenced to the mastoid electrodes average (M1–M2). EEG data were digitized at 250 Hz, band-pass filtered using a Butterworth zero-phase filter (0.01–40 Hz and 50 Hz notch filter; second order), and stored for offline analyses. Eye movements were monitored by electrooculogram (EOG) recorded by the third BrainAmp amplifier (ExG type) in bipolar modality. Horizontal EOG was recorded with electrodes over the left and right outer canthi of the eyes, while vertical EOG was recorded with an electrode pair below and above the left eye. Electrode impedances were kept below 5 KΩ. Blink and vertical eye movement artifacts were automatically corrected using the independent component analysis tool of Analyzer 2.2.2. The EEG recording was considered reliable if less than 20% of trials were rejected by an automatic artifact rejection, excluding EEG with amplitudes exceeding the threshold of ±70 µV. About 2.2% of trials were rejected.

To assess pre-stimulus activity, the EEG was divided into epochs of 1300 ms, starting 1100 ms before and ending 200 ms after stimulus onset. The baseline was applied from −1100 to −900 ms. Given that the stimulus category was unpredictable at the pre-stimulus phase, target and non-target trials were averaged.

For the intervals and electrodes to be included in statistical analysis, the “collapsed localizer” method was utilized [25]. Accordingly, a localizer ERP was obtained by collapsing (averaging) all the considered conditions. To select the analysis interval, the global field power (GFP) was calculated. The GFP describes the ERP spatial variability considering all scalp electrodes and allowing a reference-independent descriptor of the ERP. The interval in which the GPF was larger than 80% of its maximum value was used for further analysis. This approach designated a −380 to 0 ms interval from which the mean amplitude was calculated for statistical analysis. The electrodes with an amplitude larger than 80% of the maximum value in that interval were collapsed in spatial pools and considered for statistical purposes. Two foci of activity were present: a prefrontal activity (the pN) and a centro-parietal activity (the BP) component. The pN was therefore represented by a pool including AF7, Fp1, Fpz, Fp2, and AF8 electrodes (prefrontal pool). The BP was represented by a pool comprising C1, Cz, C2, CP1, CPz, and CP2 electrodes (centro-parietal pool).

### 2.3. Statistical Analysis

To assess the assumption of normality, for all measures, the Shapiro–Wilk’s W test was executed. The test showed non-significant values for any considered measures, proving their normal distributions. The Levene’s test for equality of variance was used to evaluate the assumption of homoscedasticity. This test showed no violation of homoscedasticity in the present sample. Effect sizes measured as partial eta squared (η_p_^2^) values were reported. To evaluate if changes from the pre- to post-measurements represent reliable changes, the smallest real difference percentage (SRD%) was calculated [26]. The SRD% indicates that the post-test measurement should exceed the pre-test value of that percentage to indicate a reliable change. An analysis of respondents and non-respondents to the experimental and the control training was also included. The Bonferroni correction was used for post-hoc comparisons. To measure how changes in brain activity and in cognitive performance related to basketball performance changes. For all participants, differences between the pre- and post-test in the BP, pN, RT, and FA were correlated with differences in basketball performance (mean of all tests) using the Pearson product-moment r coefficient. To classify the correlation results, the significance of each r coefficient was tested with an ANOVA comparing the correlation slope with zero. Pearson’s r coefficient was used since linear relationships between those parametric measures were expected. The overall alpha level was fixed at 0.05. All statistical analyses were performed using the Statistica 12.0 software (StatSoft Inc., Tulsa, OK, USA).

## 3. Results

In the Exp group, only two participants showed a post-test improvement of less than 10% in the basketball test, and three for the RT, FA, and ERP measures. In the Con group, no participants showed a post-test improvement larger than 10% in the basketball test. For the RT and FA and ERP measures, three participants had an improvement larger than 10%.

### 3.1. Basketball Performance Tests

**Figure 3:**
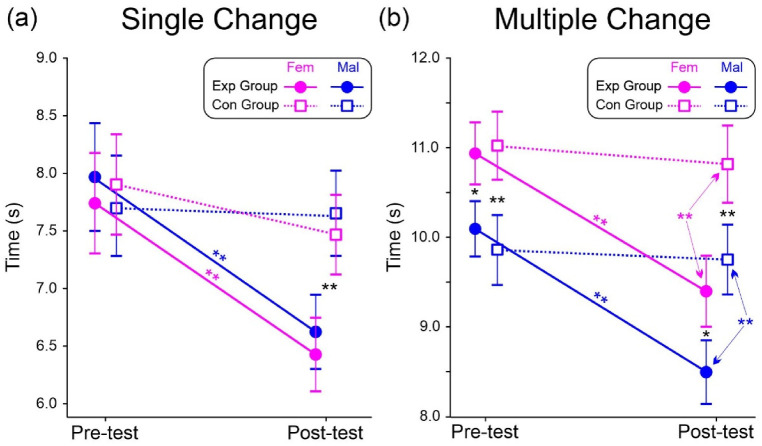
Results in basketball tests. (**a**) Single change tests completion time. (**b**) Multiple change tests completion time. Vertical bars indicate 0.95 confidence intervals. * *p* < 0.05, ** *p* < 0.01.

Table 1 presents the ANOVA results. Analysis of the single change basketball tests indicated significant group and test effects. However, the Group × Test interaction was also significant. Post-hoc comparisons showed that in the post-test the completion time of the Exp group (6.51 s SD = 0.77) was shorter (*p* < 0.001, η_p_^2^ = 0.694) than the pre-test time (7.85 s SD = 0.79, SRD% = 6.24) and was also shorter than both the pre-test (7.80 s SD = 0.75, *p* < 0.001 η_p_^2^ = 0.671) and the post-test (7.56 s SD = 0.78, *p* < 0.001, η_p_^2^ = 0.668) of the Con group. The difference between the pre- and post-test of the Con group was not significant (*p* = 0.104, η_p_^2^ = 0.106). Figure 3a shows a representation of the 3-way interaction.

ANOVA on the multiple change tests indicated a significant effect of group, test, and sex. Nevertheless, the Group × Test and the Group × Test × Sex interactions were significant too. Post-hoc comparisons substantially showed that, as for single change tests, the test was effective in the Exp group only (*p* < 0.001, η_p_^2^ = 0.698 SRD% = 6.12). In addition, males were faster than females in both pre- and post-test (*p* < 0.013, η_p_^2^ < 0.321). Figure 3b depicts the 3-way interaction.

### 3.2. Cognitive Test: Behavioral Data

**Figure 4:**
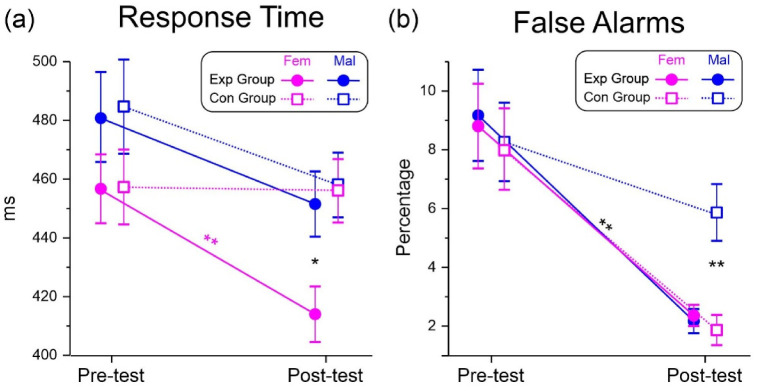
Behavioural results in the cognitive test. (**a**) Response time. (**b**) False alarms. Vertical bars indicate 0.95 confidence intervals. * *p* < 0.05, ** *p* < 0.01.

Table 2 presents the ANOVA results. Analysis of the RT showed a significant effect of the Test. The Group × Test and the Group × Test × Sex interactions were also significant. Post-hoc comparisons showed that the Exp treatment was effective in females only (pre-test = 458 ms SD = 63, post-test = 415 ms SD = 53, *p* < 0.001, η_p_^2^ = 0.635, SRD% = 8.55). The post-test Exp female RT was also faster than the males’ post-test (*p* < 0.05, η_p_^2^ = 0.383). A graphical representation of the 3-way interaction is shown in Figure 4a.

ANOVA on the FA indicated a significant effect of the Test and of the Group × Test, and the Group × Test × Sex interactions. Post-hoc comparisons showed that the Exp treatment was effective in males (pre-test = 8.1% SD = 1.2, post-test = 5.8% SD = 1.0, *p* = 0.047, η_p_^2^ = 0.288, SRD% = 9.56), and in both groups of females (Exp group: pre-test = 8.7% SD = 1.1, post-test = 2.2% SD = 0.8, *p* < 0.001, η_p_^2^ = 0.581, SRD% = 9.84; Con group: pre-test = 8.0% SD = 1.1, post-test = 1.8% SD = 0.9, *p* < 0.001, η_p_^2^ = 0.613, SRD% = 9.92). In the post-test of the Con males’ group, the FA percentage was larger than the females’ post-test condition (*p* < 0.005, η_p_^2^ = 0.416). The 3-way interaction is shown in Figure 4b.

### 3.3. Cognitive Test: ERP Results

**Figure 5:**
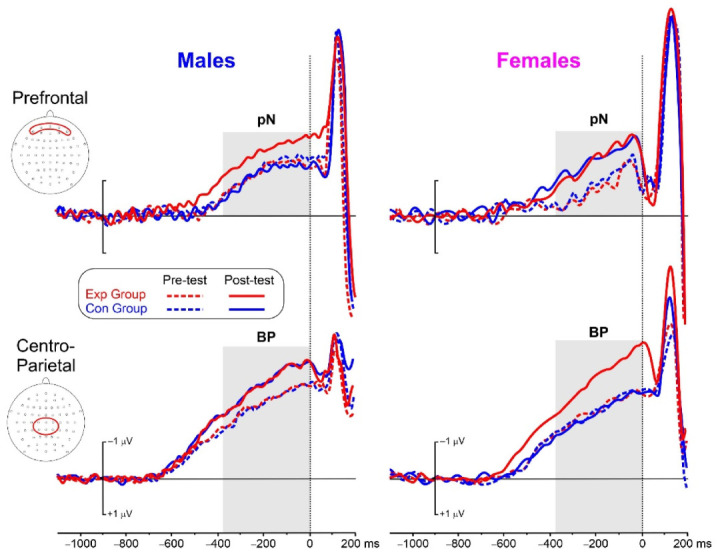
ERP waves at the medial prefrontal and centro-parietal pools of electrodes. The pools are shown in red form in the head flat-view insets. in the −320 to 0 ms interval (grey area).

Figure 5 shows the pre-stimulus ERP waveforms for the two experimental conditions (Exp, Con) in the two sex groups, before and after the training. The pN initiated around −530 ms and peaked at stimulus occurrence on medial prefrontal sites. Table 3 shows the ANOVA results.

**Figure 6:**
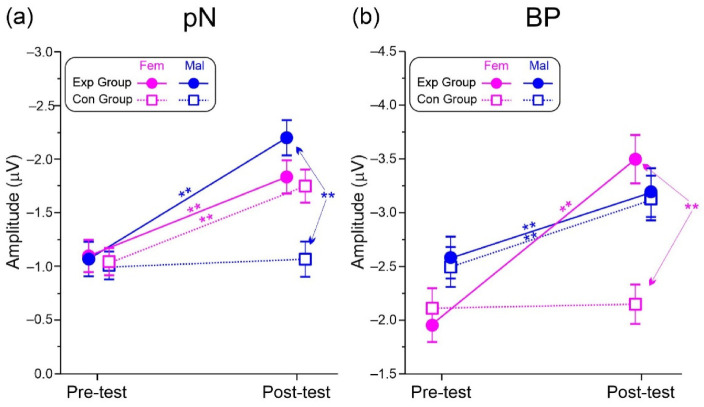
Significant 3-way interaction. (**a**) pN component. (**b**) BP component. Vertical bars denote 0.95 confidence intervals. ** *p* < 0.01.

The analysis of the pN showed a significant effect of the Test. The Group × Test and the Group × Test × Sex interactions were also significant. Post-hoc comparisons showed that both trainings were effective in all groups (*p* < 0.006, η_p_^2^ < 0.364, SRD% = 9.16) except for the males’ Con group. The pN in the post-test of the male Exp group was also larger (*p* = 0.005, η_p_^2^ = 0.402) than the males’ Con group. A graphical representation of the 3-way interaction is shown in Figure 6b.

The analysis of the BP showed a significant effect of the Test. The Group × Test and the Group × Test × Sex interactions were also significant. Post-hoc comparisons showed that both trainings were effective in all groups (*p* < 0.009, η_p_^2^ < 0.513, SRD% = 9.63) except for the females’ Con group. The BP in the post-test of the females’ Exp group was also larger (*p* = 0.001, η_p_^2^ = 0.569 than in the females’ Con group. A graphical representation of the 3-way interaction is shown in Figure 6b.

### 3.4. Correlation Analysis

Table 4 reports the results of the correlation analysis performed on all participants. Both brain activity and cognitive performance changes between the pre- and post-test significantly correlated with basketball performance. The BP showed the strongest correlation.

### 3.5. Post-Hoc Power Analysis

To verify that the ANOVA results achieved the required statistical power, the G × Power software was also used for a post-hoc power analysis. Using the effect size as the input parameter which was calculated on the η_p_^2^ for all significant effects, the analysis showed that the power (1 − β) was close to the desired power (0.95) ranging from 0.90 to 0.99. This result indicated that we used an adequate sample size.

## 4. Discussion

In the present study, we investigate possible sex differences in the effects of a cognitive-motor dual-task training intervention which is designed specifically for the sport of basketball [3] on the athletic and cognitive enforcement of adolescent semi-professional basketball players. In addition, the neural basis of these effects was also studied.

Regarding athletic performance, results confirmed our previous studies [3,4], both sexes demonstrated improvements in both single and multiple change dribbling exercises in the experimental group only. In addition, while for simple dribbling, no sex differences were found, for complex dribbling sequences, males were faster than females. This result confirms our hypothesis that males have more practice in these actions because they spend more time than females in dribbling actions [12]. In fact, Scanlan and colleagues show that female athletes performed at significantly higher running work rates with more transition movement without the ball than males, while male athletes performed significantly more dribbling. Another possible explanation came from the findings of Spierer et al. [27] investigating sex differences in lacrosse and soccer players, indicating that males, as compared with females, had faster transit speeds (i.e., time from a movement start to the end) for visual stimuli, while females tended to be faster for auditory stimuli. Another study looking at sex differences in soccer players showed that males executed significantly better than female players regarding dribbling exercise [28]. In this case, the task studied by the authors is very similar to our study where the participants had to complete the circuit with the ball as quickly as they could, changing direction by circling the eight cones four times to the left and four times to the right at varying angles. The results confirmed our theory. In general, Cheuvront et al. [29] claimed that there are sex differences in the biological structure where men have higher muscular strength and a greater aerobic capacity than females. Consequentially, the discrepancy in performance is evident. 

Behavioural results of the cognitive test before the treatment did not confirm previous studies showing faster response time in males, e.g., [13]. We found that response time was larger in females while response accuracy did not differ between the sexes. After the treatment, only the females of the experimental group became faster than all the other groups, which were stable or just tended to be faster. Response accuracy increased after the treatment in all groups except for the control group of males, which was the less accurate group. The female in the control group probably increased response accuracy because females are more capable than males in terms of cognitive control, e.g., [13]. These treatment effects partially confirm our previous study [3] which showed that both response speed and accuracy were increased in all groups, but accuracy increased more in the experimental group. This difference is probably due to the fact that Lucia et al. [3] considered male athletes only, while in the current study both the experimental and the control groups tended to be faster. The different sex composition can also explain the results of Lucia et al. [4] that mixing both males and females found a larger response time and accuracy improvements in the experimental group. Considering sex as a factor, the present study clarifies that the response time improvement following the CMDT is especially effective in females and the response accuracy improvement is especially effective in males. Considering that the CMDT cognitive enhancement was not uniform between sexes, this pattern of the result may suggest that the CMDT allows compensation for the cognitive function where each sex is weak, boosting it. To support this hypothesis early sex studies already proposed that to compensate for slower movement speed, females’ decision times should be faster and more accurate than males’ in sports [30]. This is enhanced by CMDT which simultaneously trains cognitive functions and technical fundamentals. As a result, females after the CMDT became faster than males in response times.

The sex differences in preparatory brain activity in the cognitive task seem to confirm the possible compensatory effect of CMDT. Results showed that the ERP component indexing to cognitive preparation (the pN) and associated with the response accuracy [13,17] in males is enhanced only in the experimental group. The ERP component indexing to motor preparation (the BP) and associated with the response time, e.g., [13,17,31] in females, is boosted only in the experimental group. A possible explanation of this CMDT compensatory effect can be ascribed to neuroplasticity processes compensating for cognitive functions especially lacking in a person. Indeed, the brain’s dynamic flexibility allows us to incorporate, realign or form new neural connections to adapt to new circumstances [32]; or it allows us to balance the resources of activation patterns between two mental processes, modulated by the difficulty of the task [33]. The proposed cognitive-motor dual-task training was indeed a new and highly demanding task requiring the motor and cognitive athlete’s abilities at the same time. This type of training may lead to a discrepancy between functional capacity and experience gained by the athlete, and thus may be the trigger for plastic alteration [34]. Thereafter, in male athletes, the CMDT would mainly trigger the plasticity mechanism on cognitive preparation and therefore response accuracy. In contrast, females show plasticity mechanisms mainly on motor preparation and consequently response speed.

The analysis of the responder and non-responder showed that all the participants of the Exp groups positively respond to the treatment and about 85% improved more than 10%. The SRD%, ranging from 6.2% to 10.8%, indicated that the treatment effect changes were reliable because they were within the variability of the measurement itself. In the Con group, no one showed an improvement of more than 10% (mean 2%).

The cognitive-motor dual-task protocol’s beneficial and compensatory effects are probably the result of the simultaneous stimulation of cognition and action, reinforcing the intimate relationship between the brain and movement, where the right exercise (as the proposed CMDT) not only can improve the body but also can optimize the brain (especially the executive function), e.g., [35,36,37]. This association is confirmed by the significant correlations found between brain activity and cognitive performance with sports performance. Semi-elite basketball players of both sexes might then use this type of training as a challenge to obtain technical and cognitive improvement superior to motor training alone. Therefore, taking into consideration the results of this study, coaches should include in the teams’ program individual training sessions combining exercises on a technical fundamental with cognitive exercises. Future CMDTs could be optimized from these results, suggesting that coaches should stimulate females more from a motor point of view, with drills that involve the complexity of dribbling (e.g., double change of hands) and proposed long-distance training between athletes and devices. Conversely, males should practice more on reaction time, perhaps creating special exercises on devices with limited response time also stimulating motor readiness. Moreover, CMDTs could be applied to decrease athletes’ injury risk e.g., [38], and the use of brain stimulation could be added to the CMDT to reduce mental fatigue e.g., [39].

## 5. Limitations

Some limitations of this study need to be acknowledged. First, only the pre-stimulus components of cognitive (pN) and motor (BP) preparation were analysed. Additional investigations could better support sex differences by also analysing the post-stimulus components (e.g., P1, N1, P3). Second, the current results are limited to adolescent semi-professional basketball athletes. Therefore, future studies could evaluate the possible CMDT effects on different age groups, sports, experience levels, or athletic skill levels.

## 6. Conclusions

Overall, this study showed there were sex differences in the effects of CMDT on the athletic and cognitive performance of semi-elite basketball players. Results showed that both groups had some improvements from pre- to post-tests, but after the CMDT, in single-change dribbling, male and female athletes were faster than the control group, and for multiple-change dribbling, in the beginning, male athletes were faster than females in both groups. According to the ERP results, in the Exp group of males, we found an increase in pN amplitude (associated with better accuracy); in the Exp group of females, we found an increase in BP (associated with a better response time). In conclusion, the present study confirmed the efficacy of the suggested CMDT protocol on the sport-specific as well as on the cognitive performance of semi-elite basketball male and female athletes. Additionally, it probably shows that the neurological basis for these advantages involves sex-related brain plasticity effects.

## Figures and Tables

**Table 1:** Main effects and the interaction of the ANOVA in the basketball tests. The partial eta squared (η_p_^2^) is also reported to measure the results power. Mean and standard deviation (SD) of the experimental (Exp), control (Con), males (Mal), and female (Fem) groups, and of the pre-test (Pre) and post-test (Post) are also reported and expressed in seconds. The significant effects are highlighted in red.

	Effects	F_(1,48)_	*p*	η_p_^2^	Mean (SD)	Mean (SD)
Single Change tests	Group	8.6	0.005	0.152	Exp = 7.18 (0.71)	Con = 7.68 (0.74)
	Test	114.5	<0.001	0.704	Pre = 7.83 (0.81)	Post = 7.03 (0.70)
	Sex	0.3	0.560	0.007	Mal = 7.48 (0.75)	Fem = 7.38 (0.74)
	Group × Test	55.2	<0.001	0.535		
	Group × Sex	0.3	0.568	0.007		
	Test × Sex	1.3	0.253	0.027		
	Group × Treat × Sex	2.3	0.134	0.046		
Multiple Change tests	Group	13.4	<0.001	0.218	Exp = 9.78 (0.86)	Con = 10.36 (0.88)
	Test	126.1	<0.001	0.724	Pre = 10.49 (0.89)	Post = 9.65 (0.81)
	Sex	36.4	<0.001	0.431	Mal = 9.59 (0.83)	Fem = 10.55 (0.88)
	Group × Test	82.1	<0.001	0.631		
	Group × Sex	1.1	0.294	0.023		
	Test × Sex	2.7	0.106	0.054		
	Group × Test × Sex	5.0	0.029	0.095		

**Table 2:** Main effects and the interaction of the ANOVA in the behavioural data of the cognitive test. Response times (RT) are expressed in milliseconds and false alarms (FA) as percentages. See Table 1 for other information.

	Effects	F_(1,48)_	*p*	η_p_^2^	Mean (SD)	Mean (SD)
RT	Group	1.4	0.240	0.029	Exp = 465 (63)	Con = 449 (60)
	Test	29.5	<0.001	0.381	Pre = 471 (65)	Post = 444 (60)
	Sex	4.2	0.047	0.080	Mal = 470 (66)	Fem = 447 (61)
	Group × Test	5.9	0.018	0.110		
	Group × Sex	0.5	0.462	0.011		
	Test × Sex	0.5	0.491	0.010		
	Group × Test × Sex	4.5	0.040	0.085		
FA	Group	0.3	0.574	0.007	Exp = 5.57% (1.34)	Con = 5.97% (1.36)
	Test	105.8	<0.001	0.688	Pre = 8.51% (1.48)	Post = 3.02% (0.91)
	Sex	2.4	0.128	0.048	Mal = 6.32% (1.13)	Fem = 5.21% (1.14)
	Group × Test	5.8	0.020	0.107		
	Group × Sex	1.7	0.202	0.034		
	Test × Sex	2.6	0.113	0.051		
	Group × Test × Sex	4.4	0.041	0.083		

**Table 3:** Main effects and the interaction of the ANOVA on the pN and BP components. Mean values are expressed in μV. See Table 1 for other information.

	Effects	F_(1,48)_	*p*	η_p_^2^	Mean (SD)	Mean (SD)
pN	Group	1.6	0.208	0.033	Exp = −1.48 (0.26)	Con = −1.26 (0.22)
	Test	92.9	<0.001	0.659	Pre = −1.18 (0.21)	Post = −1.66 (0.28)
	Sex	0.4	0.515	0.008	Mal = −1.32 (0.25)	Fem = −1.43 (0.23)
	Group × Test	24.9	<0.001	0.341		
	Group × Sex	2.3	0.134	0.046		
	Test × Sex	0.1	0.816	0.001		
	Group × Test × Sex	9.3	0.004	0.162		
BP	Group	0.8	0.368	0.017	Exp = −2.89 (0.46)	Con = −2.65 (0.44)
	Test	32.0	<0.001	0.400	Pre = −2.47 (0.44)	Post = −3.07 (0.55)
	Sex	0.4	0.502	0.009	Mal = −2.86 (0.48)	Fem = −2.68 (0.47)
	Group × Test	8.3	0.005	0.147		
	Group × Sex	0.5	0.459	0.011		
	Test × Sex	>0.1	0.968	0.001		
	Group × Test × Sex	8.2	0.006	0.146		

**Table 4:** Correlations between brain activity (BP and pN) and cognitive performance (RT and FA) with global basketball performance (basket).

Pre Minus Post	r	*p*
BP vs. Basket	0.412	0.008
pN vs. Basket	0.389	0.018
RT vs. Basket	0.332	0.023
FA vs. Basket	0.319	0.031

## Data Availability

Data are available from the corresponding author upon request.

## Supplementary Materials

The following supporting information can be downloaded at: https://www.mdpi.com/article/10.3390/brainsci13030443/s1. Supplementary Video Material: Video Motor Training and Video Cognitive-Motor Training.
